# Building biodiversity in neighbourhood parks in Bangalore city, India: Ordinary yet essential

**DOI:** 10.1371/journal.pone.0215525

**Published:** 2019-05-06

**Authors:** Savitha Swamy, Harini Nagendra, Soubadra Devy

**Affiliations:** 1 Ashoka Trust for Research in Ecology and the Environment (ATREE), Srirampura, Jakkur Post, Bangalore, Karnataka, India; 2 PES Campus Pixel Park, Electronics City, Bengaluru, Karnataka, India; Sichuan University, CHINA

## Abstract

Cities comprise of mixed green patches that vary in size and are highly scattered and disconnected. Although small green spaces largely dominate the cityscape, they are often neglected and ignored by the naturalists and conservationists, as they do not fulfill the large green spaces criteria. The citizens on the other hand seem to have a different perception and requirements from small green spaces as they are within their neighbourhood. Bangalore, a developing city within South India, consists of a large number of newly formed residential areas which have pocket green spaces in the form of neighbourhood parks (henceforth NPs). They are maintained by the municipality and are mainly designed for recreation purposes, completely neglecting the fact that these spaces could be essential for biodiversity. Here, there is a disconnect between the requirements of the citizens, conservationists and the end product that the municipality delivers. Here, through a questionnaire survey we assess the biodiversity citizens are fond off, and use them as surrogate taxa for the not so immediately obvious taxa, insects to enumerate the biodiversity within NPs. We analyze and identify landscape characteristics around NPs which could enhance the biodiversity within NPs. Our results reveal that people are fond of Birds and Butterflies and we use them as surrogates for the inconspicuous taxa to assess biodiversity within NPs. 55 tree species, 45 species of birds, 41 species of butterflies and 68 morpho species of insects were recorded. We demonstrate that small green spaces are critical systems and help support biodiversity across three scale within the city. Interestingly, results suggests that density of NPs is more important rather than the size of NPs. Also, the presence of high density of NPs within a neighbourhood could support similar biodiversity that large green spaces support. Finally, this study provides insights on the landscape matrix that could help enhance biodiversity support service within NPs and the surrounding neighbourhood.

## Introduction

Fragmented and isolated habitat patches are the signature landscape of most cities [1]. These habitats are heterogeneous and can vary from small home gardens to large sprawling campuses and heritage parks. Regardless of the size, they support biodiversity and provision a range of ecosystem services to the community [2–4]. Although large green spaces (henceforth LGS) support more biodiversity, the smaller patches surrounding them help connect the LGS which are otherwise isolated due to the built-up environment, additionally providing resources and habitats for various taxa. The role that these small green patches play often goes unnoticed and neglected as LGS provide more services, support more biodiversity and comprise more of natural vegetation [5]. Neighbourhood parks (henceforth NPs) are pockets of green spaces within cities. Although NPs, are small and may not support biodiversity on their own, they could be acting as stepping stones, there by facilitating movement for vagile taxa such as birds, butterflies and insects, resulting in an increase in biodiversity at the regional scale [6–8]. NPs can serve as vital spaces for biodiversity that could strengthen the health of the ecosystem and the services provisioned to the society [9,10] especially at the neighbourhood scale. Therefore, it is essential to understand the landscape features and habitat complexities that may affect biodiversity within and around these small habitat patches.

Over the recent years, cities worldwide are undergoing dramatic transformation. This has resulted in improved infrastructural facilities but poorer environmental conditions, mainly because they lack in the planning of green spaces within cities. Bangalore city, which was once regarded as “Garden City” has now lost most of its gardens and green spaces and has transformed into the “Silicon City” of India. Gardens which used to be in large numbers and comprised prominently of local plant species, highly stratified have now been simplified intomore open landscapes dominated with exotic plant species [11,12]. This has altered not only the landscape and eroded the biodiversity but has also changed the attitude of the community towards green spaces neglecting and undervaluing the services that small green spaces such as NPs provide [5].Interestingly, over the last decade in most developed countries, there are indications that urban green spaces are valued from being spaces that not only provide recreational services but also serve as biodiversity conservation areas [13]. This shift could help enhance biodiversity conservation within NPs with the involvement of the community. We evaluate the role of NPs as critical green spaces for supporting biodiversity within Bangalore city. Also, this study assesses if people’s preferred taxa can be used as surrogates for the inconspicuous taxa such as insects. Also, we analyze if the larger surrounding habitat requirement across taxa can provide insights into understanding the factors that could help enhance biodiversity services within NPs and the neighbourhood. Thus, this paper builds on to discuss ways of developing green networks within dominant residential landscape configurations, which could be sources of biodiversity within cities.

## Methods

### Ethics committee approval process

As of now ATREE does not have an ethics committee.

The proposal for this study was reviewed by doctoral advisory committee (DAC) prior to field data collection. DAC members Dr. Harini Nagendra, Dr. Seema Purushothama and Dr.Soubadra Devy approved the study and the questionnaire was trial run n the field before it was finalized to avoid any questions which may be sensitive to respondents. In addition, the synopsis of the study was presented at ATREE and the audience included social scientists, developmental economist, interdisciplinary experts and others.

Permission from the ward officer from the Horticulture Department Bangalore were taken to work in the parks.

### Study area

Neighbourhood Parks in Bangalore city were selected using the Bangalore Comprehensive Development Plan [14]. The city is bounded within three zones/belts and further divided into 48 planning districts (henceforth residential areas). The first belt is Bangalore Core, also called ‘‘petta” comprises the old residential and commercial areas; second belt comprises the old residential areas and the third belt comprises newly developed or peri-urban areas.

**Fig 1:**
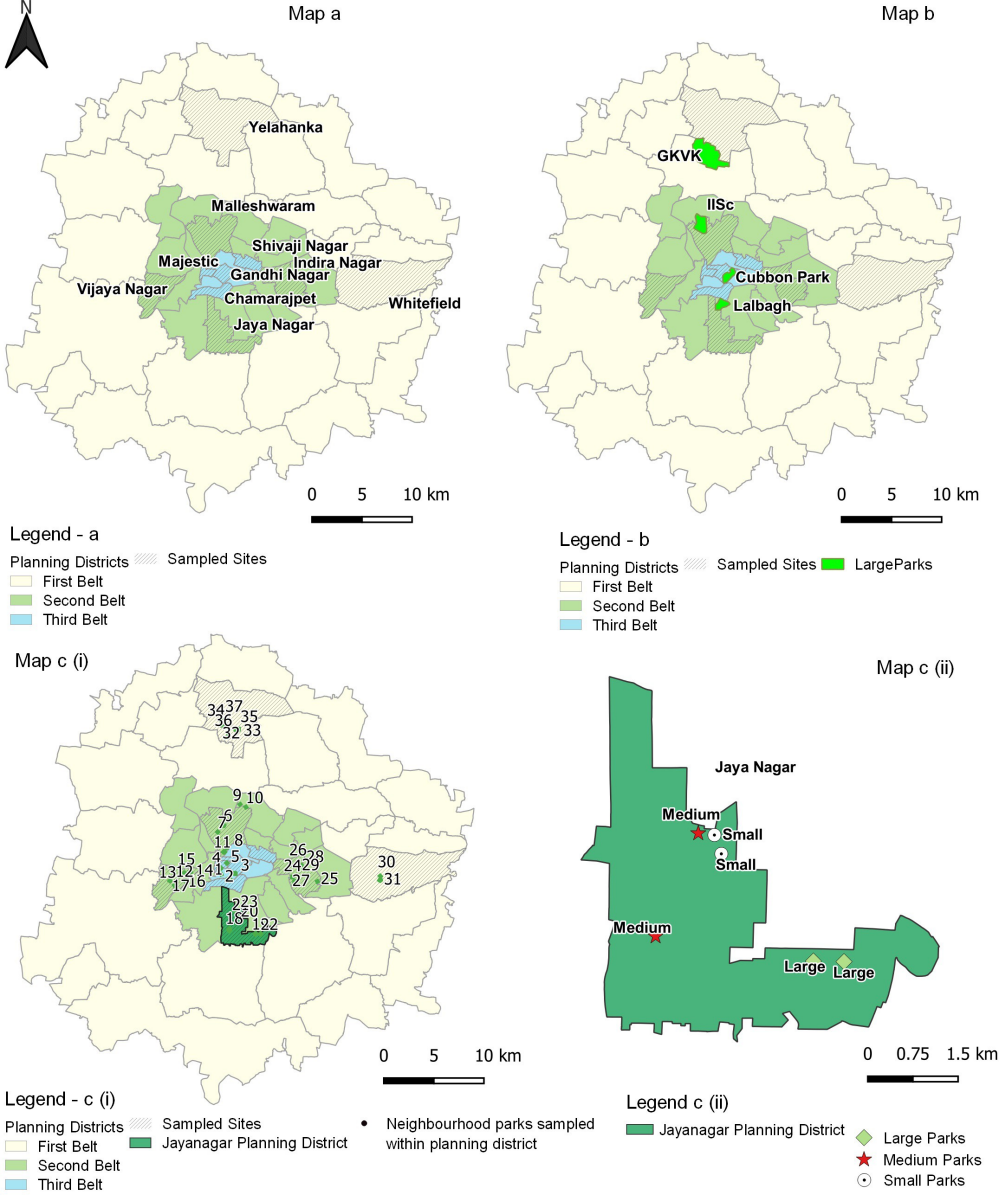
Map of Bangalore (A) With 10 districts sampled across the three zones; (B) Location of the four large green spaces sampled across Bangalore; (C) Map a: Distribution of the 37 Neighbourhood Parks sampled across the city; Map b: close up of Jayanagar District and the NPs distribution within the district.planning.

Based on cardinal directions, 4 residential areas in each zone were sampled. The third zone is an exception, where only 2 areas were sampled, as other areas in the zone were under development. A total of 10 residential areas were chosen for this study (Fig 1A). An exploratory survey within the chosen residential areas helped to categorize NPs into three size classes: Small–300 to 1000 sq m; Medium–1000 to 5000 sq m and large ≥ 5000 sq m). The survey revealed that distribution of NPs was highly variable across the city, from a few residential areas possessing numerous NPs to a few with just one or no NPs at all. To standardize parks across all areas, the following was done: All parks within the 10 residential areas were surveyed and six NPs (henceforth focal parks) were sampled per residential area, two replicates representing for each size categories. The other unsampled parks within the residential areas termed non-focal parks. Thus, a total of 37 NPs (small–11; medium–14; large–12) were chosen for the study, as some residential areas did not have representations of all size classes (Fig 1B). Also, four LGS in the city: Cubbon Park and Lalbagh (heritage parks comprising wooded areas, open area with grass and water body), Indian Institute of Science (IISc) and Gandhi Krishi Vigyana Kendra (GKVK) (institutional campuses, which have mixed vegetation, from highly wooded areas to highly maintained manicured areas); were selected and mapped in conjunction with NPs (Fig 1C). This was to assess if NPs are influenced by these LGS and to determine if there is decay in nestedness of birds and butterfly species with increasing distance from the LGS across taxa.

#### Biodiversity survey

**Fig 2:**
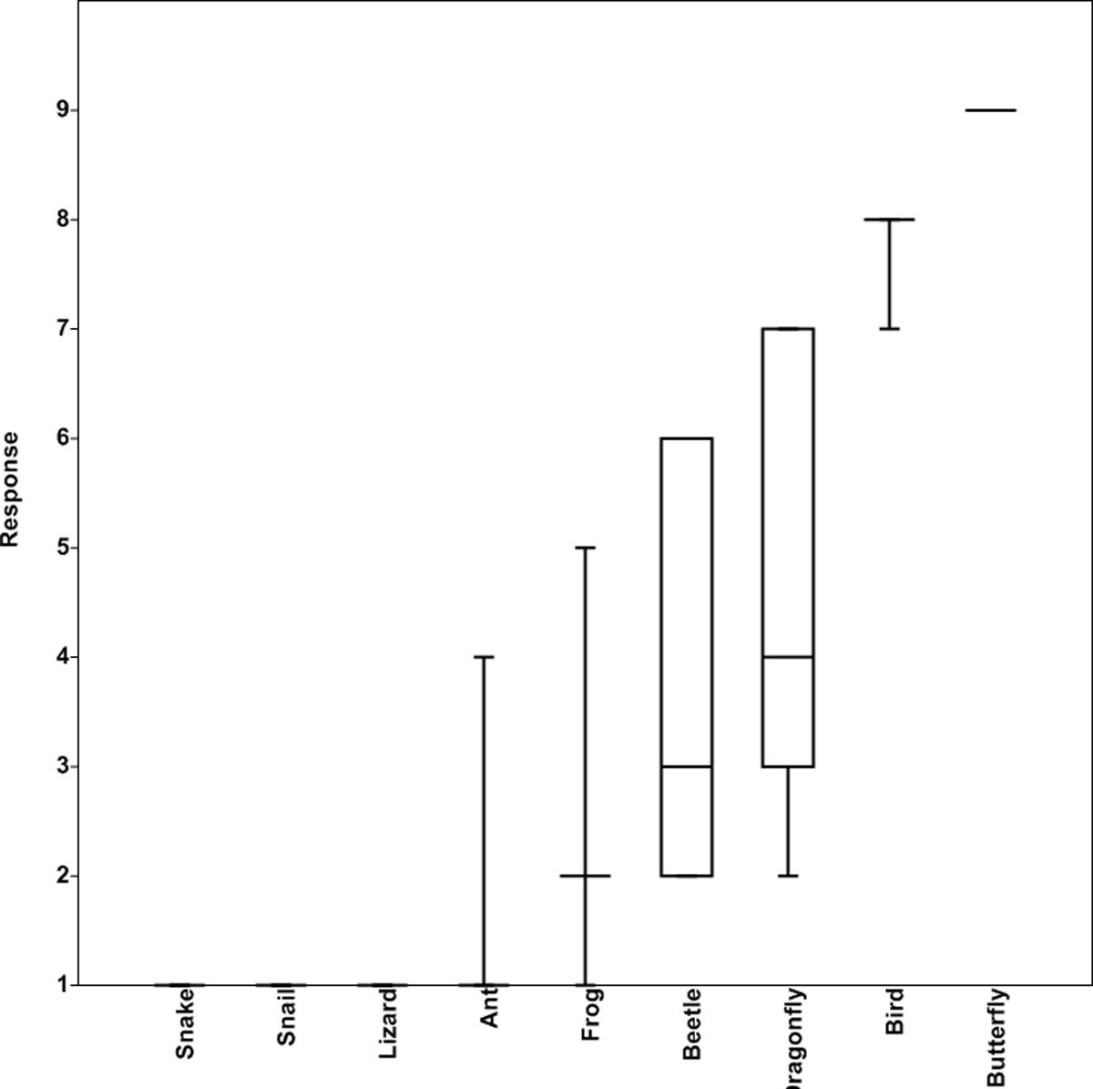
Likert-scale fondness survey across nine commonly found taxa within neighbourhood parks using box and whisker plot.

A ‘‘biodiversity fondness” survey was conducted and Likert-scaling method was employed to assess people’s tolerance levels towards nine commonly encountered taxa in NPs. A 9-level scaling method [15] was used, where 9 denoted fondness towards the taxa and 1 indicated complete intolerance. A total of 563 surveys conducted amongst park users, helped identify the two taxa ‘‘birds and butterflies” that people are fond of and these were then sampled in all the 37 NPs using standard ecological methods. This also helped determine if taxa which emerged as high ranking in the fondness survey, served as a surrogate of all other inconspicuous taxa that were present in the park (Fig 2).

#### Surrogate taxa (birds and butterflies) survey

Survey on birds and butterflies were conducted using point count method [16]. The main factor that determined the number of points to be used for sampling the park was the observation radius for recording birds and butterflies. The average width of the park determined the observation radius across all parks. The sampling points depended on the size of the park as follows: small–1 point; medium–2 points and large–3 points. Observation time at each point was approximately 10 minutes with an interval of 5 minutes between two sampling points and each point was sampled thrice. Birds and butterflies within 5 m radius of the point was recorded. The sampling time was staggered on basis of the activity period of the taxa. Sampling of birds was carried out between 6:30 and 8:30 am and butterflies were sampled between 9:00 and 11:00 am. Sampling on cloudy, windy and rainy days was avoided. The survey was conducted for two consecutive years, 2009 and 2010, and each park was sampled during three different seasons (March to May–Summer; June to August–Monsoon; November to February–Winter). Birds heard and on flight were not recorded.

#### Inconspicuous taxa survey (insects)

Pitfall method was carried out to sample ground insects within NPs. The number of pitfalls laid in each park was again determined by the size of the park: small–2, medium–4 and large–6 pitfalls were placed in the park and were left undisturbed for 24 hours. Pitfall traps containing soap water were buried on ground until the rim. The following day, insects within each pitfall were removed and stored in separate vials containing 70% alcohol for identification in the laboratory. All insects collected were identified up to morpho species [17].

#### Vegetation survey

NPs comprised trees, shrubs and herbs and were sampled on basis of the size of the park. Distribution of trees across parks varied: only along the boundaries, only in one specific area to being randomly scattered all across the park. Since, there was a variation in tree distribution, quadrat sampling method for trees could not be employed as it would result either in over sampling or under sampling. Also, an *a priori* sampling study showed that the species accumulation curve for trees plateaued at 10 trees for small parks, 20 in medium and 30 in large parks. Hence, trees were sampled as mentioned above. Sampled trees were identified up to species level and parameters such as girth at breast height (GBH) and height were recorded. Shrubs were sampled using a 5 × 5 m quadrat and herbs were sampled using a 1 × 1 m quadrat. Quadrats for herbs were nested within the quadrats that were laid for shrubs. The number of quadrats which were placed for shrubs and herbs were again determined by the size of the park: small–2 quadrats, medium–4 quadrats and large–6 quadrats. Densities of shrubs within the quadrat and proportion of herb vs. grass were calculated. Unidentified species were photographed or collected for later identification.

#### Habitat characterization

Four habitat characteristics of NPs were recorded. Canopy cover was assessed through digital photographs taken at shoulder height to capture the canopy cover within each NP [17]. Size of the park determined the number of capture points for assessing the canopy cover of the park: small–2 points, medium–4 and large park–6 points. The photographs were later gridded using image editing package Photoshop TM and the number of filled vs. empty cells determined the percentage canopy cover within the NPs [17]. Landscape characteristics such as tree distribution, percentage of open space were considered key features that could influence the biodiversity within NPs. Hence, these categories were identified as compact landscape type–trees distributed along the boundary and densely (8 to 10 trees) scattered in the centre of the park; mixed landscape type–trees along the boundary and a few (3 to 4 trees) in the centre and open landscape type–trees present only along the boundary of the park. Also, to analyze habitat complexity (henceforth HC), a combination of parameters such as vertical stratification, height diversity and openness of vegetation along with diversity of plant species were considered. Existing HC formulae did not consider multiple habitat parameters, hence a formula was derived [18–20]. This formula encompassed vegetation parameters within NPs, to analyze if HC as a whole within the NP influenced the selected taxon.

HC value was derived using the following formula:

$$HC\;=\;\sum \left\{\left[\left(\frac{1}{\left(1-\left(\frac{Tn}{TN}\right)\right)}\right)+\;\left(\frac{Tab}{TA}\right)+\;\left(\frac{T\sigma }{TM*100}\right)\right]+\;\left[\left(\frac{1}{\left(1-\left(\frac{Sn}{SN}\right)\right)}\right)+\left(\frac{Sab}{SA}\right)\right]+\;\left[\left(\frac{1}{\left(1-\left(\frac{Ln}{LN}\right)\right)}\right)+\left(\frac{1}{Lp}\right)\right]\right\}$$

T = Trees, S = Shrubs, L = Lawn

*n* = species richness; *N* = total species richness across all parks; *ab* = abundance; *A* = area of park (small park = 1; medium = 2 and large park = 3); ***σ*** = standard deviation for tree height; M = Mean of Tree height; Lp = proportion of the lawn

Prior to applying the HC equation, the variables were normalized using the formulae for individual parameters, which are mentioned in parentheses for deducing habitat complexity values for individual NPs.

Often, size of NPs may be a constraint to incorporate a combination of all vegetation features and landscape elements that are suitable for multiple taxa within a single park. Hence, identifying specific variables for each taxa might be an alternative option, which could help implement a few features across a set of NPs within a neighbourhood so as to increase local species richness. Individual habitat variables such as canopy cover, tree abundance, shrub and lawn proportion may exhibit differential influence, which could enhance the diversity of either birds, butterflies or insects within NPs (henceforth park scale), which were identified using the hierarchical partitioning (henceforth HP) method [21]. To increase normality, tree and shrub abundance data were log transforming while proportions such as canopy cover, shrub, lawn and herb coverage were arcsine transformed [22]. Non-metric multidimensional scaling (NMDS) was carried out to eliminate correlated variables. This was followed by *Z*-score analysis. Based on 100 permutations, habitat variables such as canopy cover, built-up area, with values higher than 1.65 which indicate significance at the 95% confidence level, were considered an important influence on the response variable [7,23]. The HP helped identify the independent influence of each variable on the three sampled taxa.

Morisita-Horn Similarity was used to compare the distributional patterns of actual assemblages of species for all three taxa across geographical locations. Morisita–Horn Index with abundance data was used, and values that were <30% suggested dissimilarity and >60% similarity for individual taxa. Also, Jackknife estimator was carried out to evaluate if there was any relationship between species richness and size class of NPs for all the three taxa.

#### Landscape effects

Landscape at the neighbourhood scale may influence ecological processes that in turn can have an effect on local biodiversity of mobile taxa such as butterflies and birds [24,22,25,26,27,10]. Relatively sedentary taxa such as ants and other litter insects could be more influenced by local habitat structure at an individual park scale. Therefore, based on the vagility of the species, the influence of surrounding landscape was determined using buffers of various radii. A 0.5 km buffer was used for insects and 1 km for butterflies. The imagery size procured allowed only for a maximum of 5 km radius buffer for birds. HP analysis helped identify key individual variables in the surrounding matrix (henceforth neighbourhood scale) of NPs, which could contribute to the biodiversity of NPs as well.

To analyze the landscape features around NPs, Geo Eye (3/3/2009), Quick Bird (26/02/2009) and IRS LISS III images (14/03.2009) were used. The resolution of Quick Bird and Geo Eye images was 3 m and IRS LISS III was 23 m. Also, Quickbird and GeoEye was consistently used for the analysis of insects and butterflies. The IRS images were used only for the larger spatial scale analysis of birds. The different resolution of the different sensors had no effect on the analysis. These images were used for supervised classification in IDRISI Taiga. The five land-use categories that were identified: green area–comprised parks, avenue trees; built-up area–comprised building, road and other grey developed areas; open spaces–barren land, open fields without vegetation; water area–water bodies; in the administrative units within zones 1 and 2, and an additional classification of agriculture and plantation in units within zone 3.

Landscape features such as green vs. built-up area were analyzed at three scales–park scale, neighbourhood scale and city scale. Buffers of 1 km for butterflies and 5 km radius for birds were created around individual NPs at the park scale; buffer of 2 km for both taxa at the neighbourhood scale and a 5 km merged buffer, which represents several residential areas at the city scale. In case of the 5 km buffer, if two or more parks overlapped, the buffers were merged, thus forming clusters, which represent many residential areas within a geographical location, termed localities.

Also, the four LGS in the city, Cubbon Park, Lalbagh, IISc and GKVK were mapped in conjunction with NPs. Constraints on the extent of the imageries allowed for incorporating only these four LGS for this study. This was to assess if NPs are influenced by these LGS and to determine if there is decay in nestedness with increasing distance from the LGS across taxa. Bird and butterfly checklists of these LGS were collated from naturalists and experts within the respective institutions for the period that coincides with this study period. To assess if NPs support a nested subset of the species richness seen within the selected LGS, buffers of 1 km and 5 km for butterflies and birds, respectively, were created. The pooled assemblages of birds and butterflies of NPs at varying distance buffers were then listed against the checklist obtained for individual LGS to assess the proportion of the volant taxa butterflies and birds that NPs supported.

## Results

### Diversity

**Fig 3:**
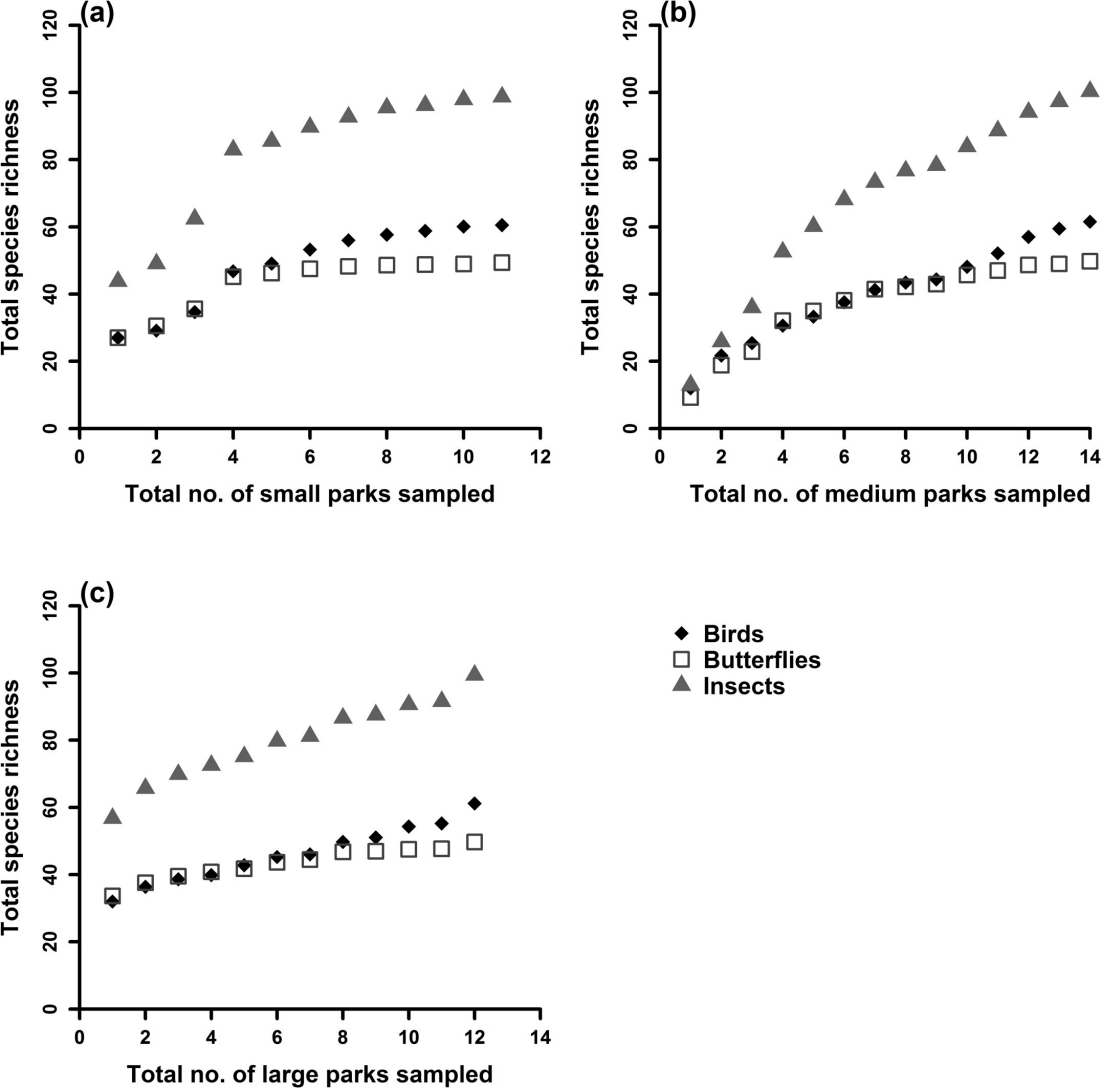
Jackknife estimation for species richness across size class of NPs: (a) Small parks; (b) Medium parks and (c) Large parks.

**Fig 4:**
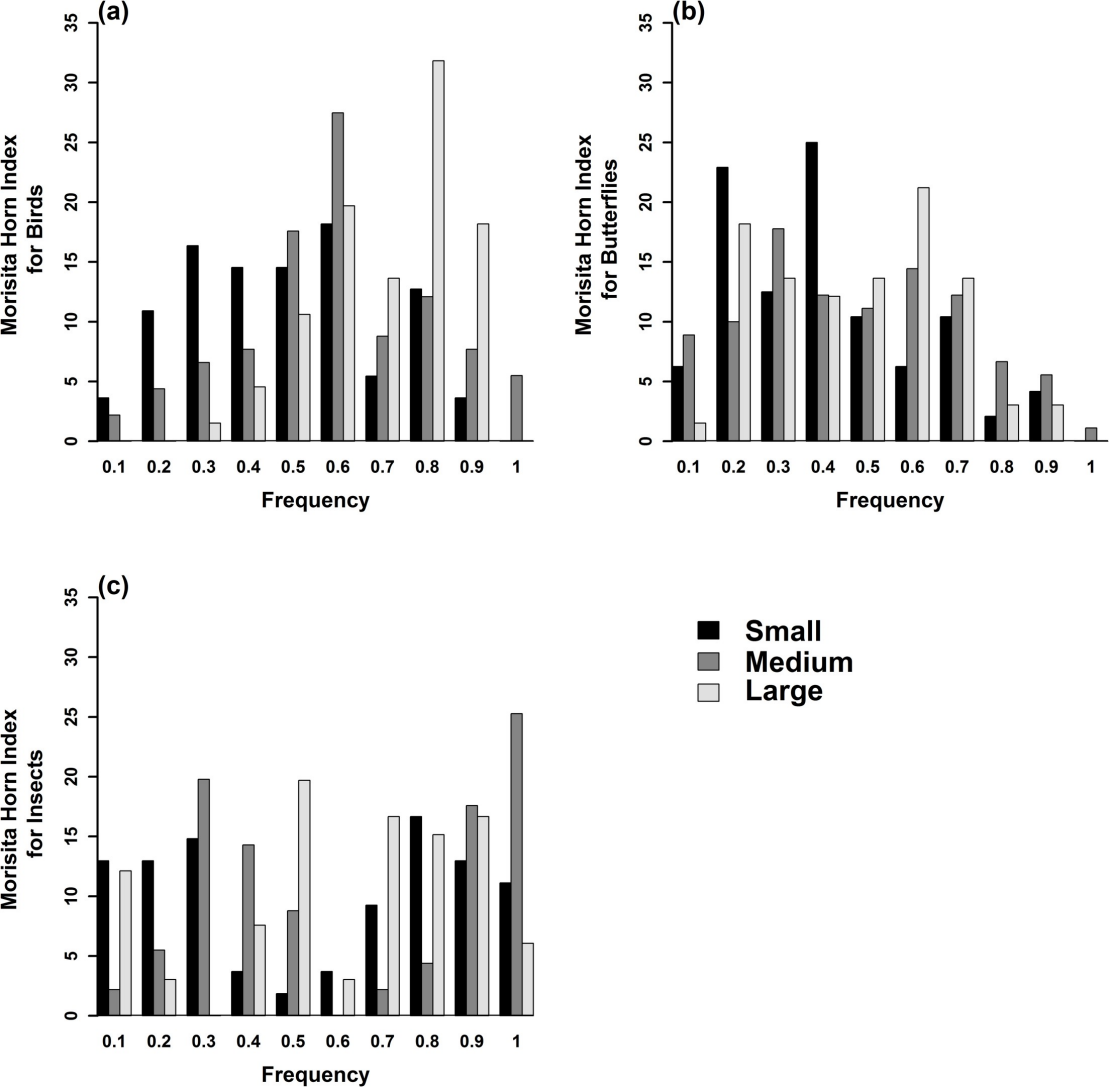
Frequency distribution of Morisita-Horn index values across size class of NPs: (a) Birds; (b) Butterflies and (c) Insects.

A total of 55 tree species, 45 species of birds, 41 species of butterflies and 68 morpho species of insects were recorded from a survey of 37 NPs in Bangalore. The frequency distribution graph showed that there is a significant difference in size class of trees (*F* = 14.68, *p* = 6.09*E* – 07) and that large and medium parks have a wider tree size class distribution than small parks (*t* = 5.42, *p* = 0.01). Tree data showed that there is equal representation of native and exotic species across NPs. Regression analysis indicated that both abundance and species richness of birds, butterflies and insects increased with size of the park (Table 1). There is a weak linear relationship between park size and species abundance/richness in some cases explaining only 13% of variance in the empirical data (bird richness). This is important, presumably, as it indicates the likely importance of other variables such as habitat composition or landscape variables. Jackknife estimator for species richness showed that large parks are species rich compared to medium and small parks (Fig 3). Using abundance data, frequency distribution of Morisita–Horn index values across the three size classes of NPs showed that only small parks are dissimilar to medium and large parks with respect to the three taxa they support (Fig 4).

**Table 1:** Pearson's correlation between species richness/abundance with size of NPs across taxa.

Taxa	Species Richness/Abundance vs Size	R and p values
**Bird**	Abundance vs size	0.4770, 0.01*
	Species richness vs size	0.3655, 0.05*
**Butterfly**	Abundance vs size	0.4744, 0.01*
	Species richness vs size	0.3817, p = 0.02*
**Insect**	Abundance vs size	0.4196, p = 0.01*
	Species richness vs size	0.518, p = 0.01*

### Response of habitat variables to scale

#### Park scale

**Fig 5:**
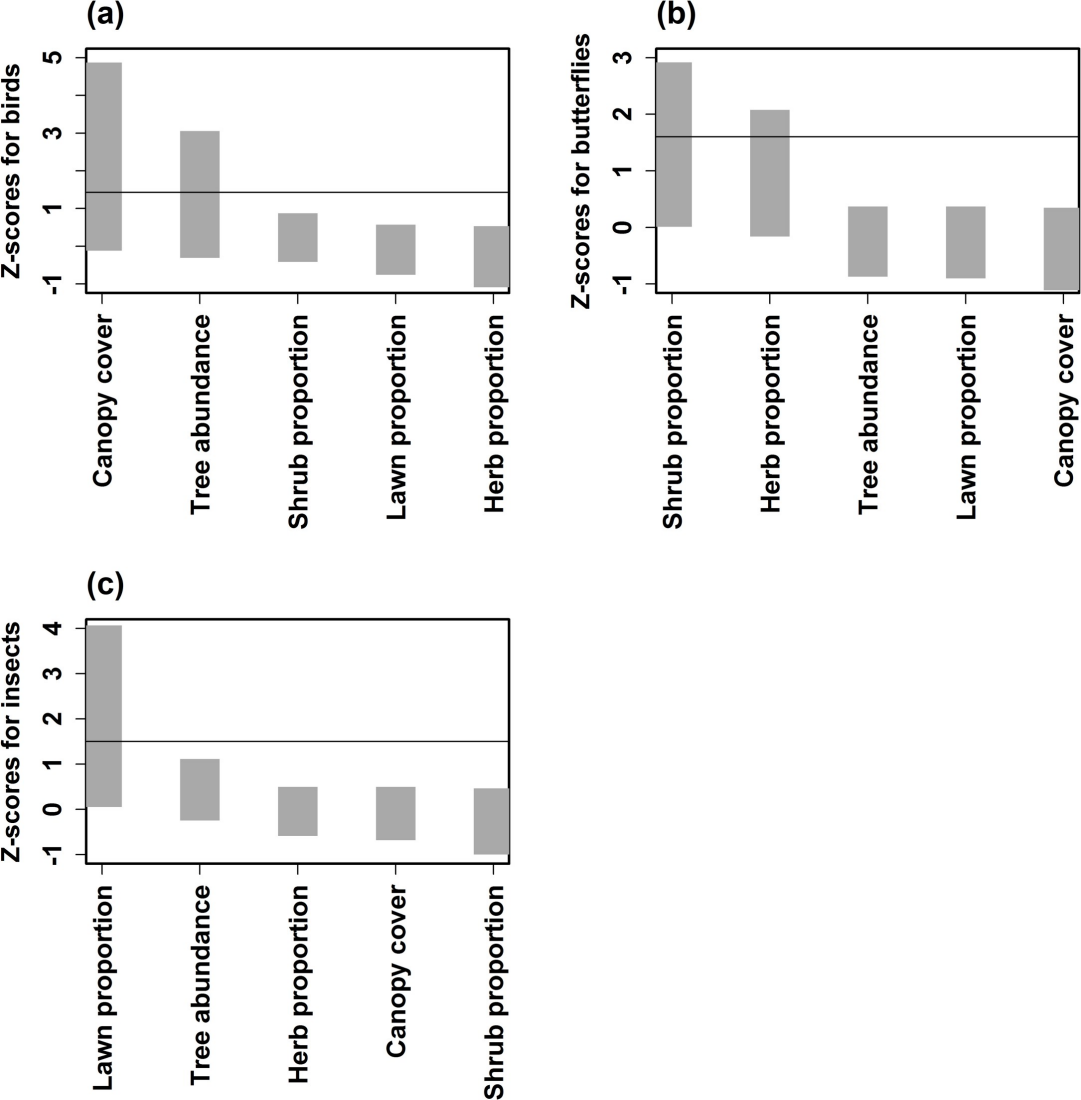
Hierarchical partitioning to determine variables that influence (a) Birds; (b) Butterflies and (c) Insects at park scale.

Some common and abundantly found tree species in all parks were *Grevillea robusta*, *Polyalthia longifolia* and *Bauhinia sp* and shrubs were *Durantha*, *Croton* sp. and *Hamelia patens*. Landscape type analysis of NPs, showed that 75% of the parks sampled were of the open type, 18% mixed and 5% of the compact type. Habitat had a differential influence on the three taxa, canopy cover and tree density influenced bird species; the proportion of shrubs and herbs present in NP was critical for butterflies and insect species were largely influenced by the proportion of lawn in parks (Fig 5). Similarly, bird, butterfly and insect abundance, and species richness, were significantly related to the habitat complexity of the NP except for butterfly species richness (Table 2).

**Table 2:** Regression analysis of habitat complexity values across all three taxa within NPs.

	R value	p value
Bird species richness	0.3738*	0.05
Bird abundance	0.3462*	0.05
Butterfly species richness	0.2341	0.05
Butterfly abundance	0.3355*	0.05
Insect species richness	0.3174*	0.05
Insect abundance	0.4101*	0.02

*indicates values which are significant at p value 0.05

#### Neighbourhood scale

**Fig 6:**
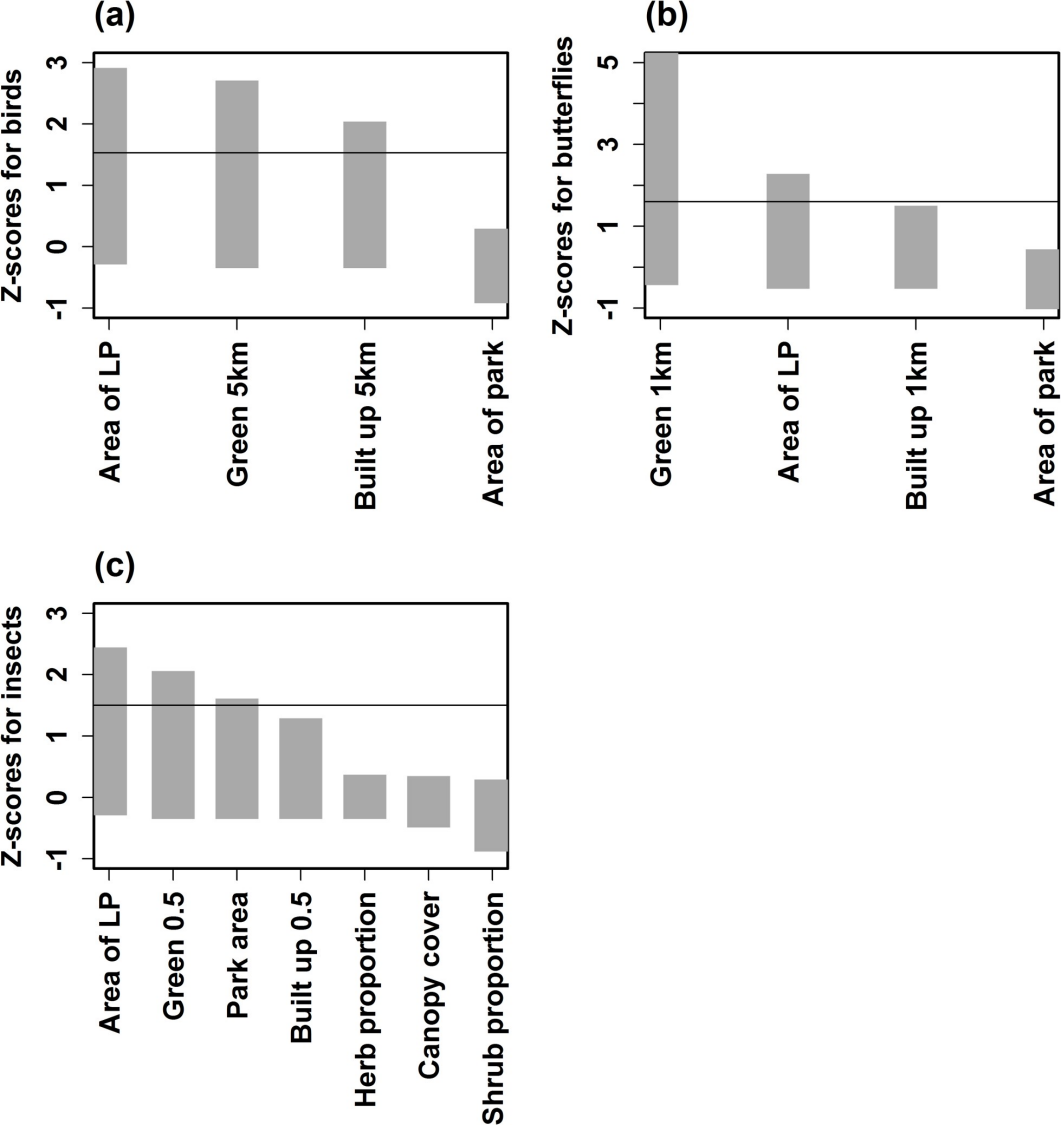
Hierarchical partitioning to determine variables that influence (a) Birds; (b) Butterflies and (c) Insects at neighbourhood scale.

Analysis of key surrounding landscape variables of NPs showed that species richness of birds was influenced by cumulative area of large green space, ratio of green area and built-up area (sq km), over a radius of 5 km (Fig 6A). For butterflies and insects, green area at the localized scale, which comprised home gardens, avenue trees and other satellite NPs within 0.5 km and 1 km, are important determinants of species richness (Fig 6B and 6C).

#### City scale

**Fig 7:**
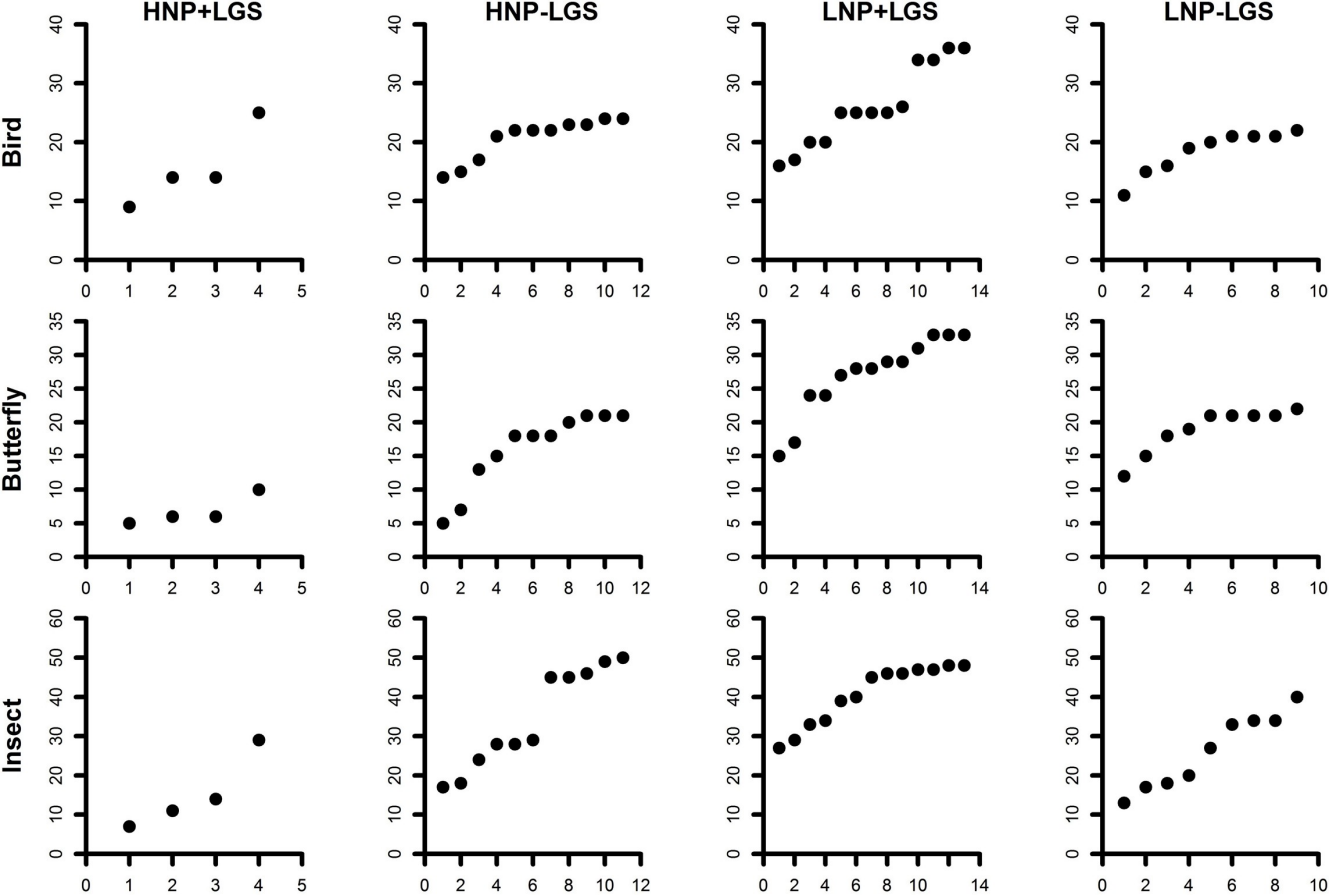
Species accumulation for three taxa across varying density of NPs at city scale.

Based on the non-focal and focal NPs, the density of parks, NPs were further characterized into four distinct groups as high-density NPs (henceforth HNP) along with the presence/absence of LGS (HNP; HNP + LGS; HNP–LGS) and low-density NPs (henceforth LNP) in presence/absence of LGS (LNP, LNP + LGS; LNP–LGS). Species accumulation across varying NP densities within a 2 km buffer, showed that high-density packing of NPs in the absence of LGS (HNP–LGS) and low density of NPs in the presence of LGS were high in species richness (LNP + LGP; Fig 7).

Collated bird and butterfly checklists, from focal NPs at varying distances from LGS, showed that there is no decline in bird species richness. However, for butterflies, there was an increasing decline in species richness with increase in distance from LGS (Table 3).

**Table 3:** Contribution of NPs to the faunal diversity within LGS.

Taxa	Dist from LGS	GKVK		IISc		Cubbon Park		Lalbagh	
		No. of NPs	Propn.*	No. of NPs	Propn.	No. of NPs	Propn.	No. of NPs	Propn.
Butterflies	1km	3	8.3	2	22.4	3	36.7	0	NA
	5km	6	12.6	9	23.45	21	41.1	21	33.67
Birds	1km	3	6.8	2	66.6	3	9.5	0	NA
	5km	6	9.9	9	77.7	21	44.6	21	54.8

* abbreviation of proportion of fauna from the large space that are represented in NPs

**Fig 8:**
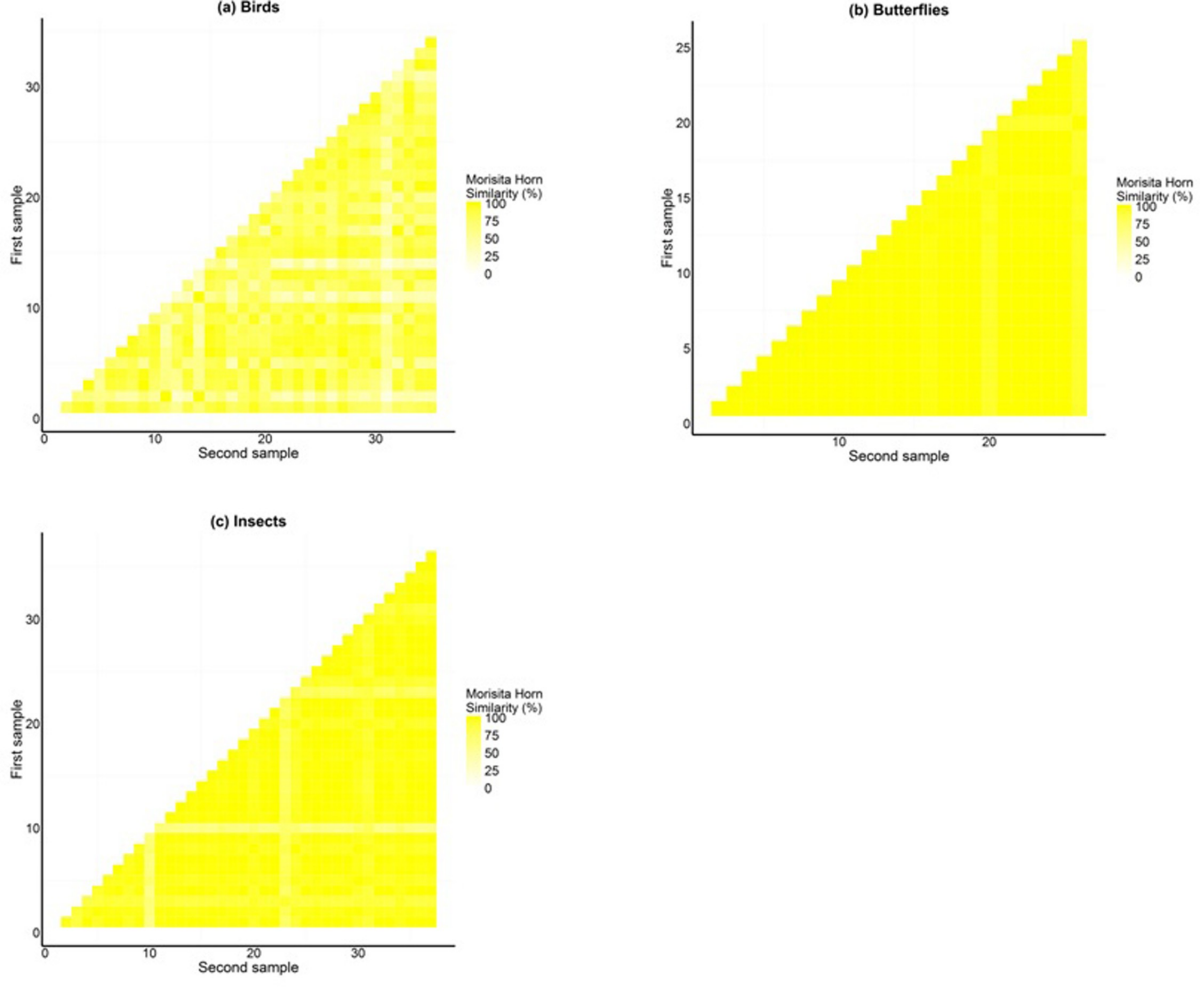
A visual representation of Morisita-Horn index indicating species assemblages across 1. Park scale for a) Birds; (b) Butterflies and (c) Insects.

We compared the distributional patterns of actual assemblages of species for all three taxa across localities. Morisita–Horn index with abundance data using extremities <30% dissimilar and >60% similar, for individual taxa indicated homogeneous pattern across all three taxa (8A, Fig 8B and 8C).

## Discussion

Habitat determinants within the park could be the key factor for local species richness than size [28].The study showed that habitat characteristics that influenced each of the taxa varied and almost all showed only moderately linear relationships. Large NPs have a higher representation of birds, butterflies and insects in comparison to medium and small NPs. Results also showed that among the habitat variables—the canopy cover for birds, proportion of shrubs for butterflies and proportion of lawns for insects showed some influence on their presence. Other studies have also shown plant diversity at a local scale for butterflies; canopy cover, presence of trees, shrubs for birds and leaf litter for insects could influence their local species richness [22,29,30,31,17]. Even overall habitat complexity showed moderately linear relationship with all the 3 taxa. In general, there was a lack of very strong relationship with habitat variables that were examined at the park scale. This probably suggests that the immediate landscape matrix surrounding the NPs could have a major influence on the biodiversity. High density of small NPs determined the butterfly diversity in the absence of LGS. Implying that in a highly fragmented landscape, butterflies are more dependent on the density of green spaces at the neighbourhood scale. Suggesting that along with microhabitat composition and heterogeneity within NPs, the neighbourhood matrix within which NPs are embedded plays a crucial role in determining the local biodiversity. Birds are dependent on LGS, however, we recorded the Greenish Leaf Warbler (*Phylloscopus trochiloides*) and Blyth’s ReedWarbler (*Acrocephalus dumetorum*) which are migrant bird species, within neighborhoods which comprised several unmaintained vacant sites comprising wild vegetation surrounding the NPs. Neighbourhood green spaces with unmanaged vegetation such as tall grass, shrubs and thick bushes could be essential to support local biodiversity [32]. The migrant bird species were recorded within the second belt, which comprised of LGS, several tree lined avenues and large number of NPs. Presence of street-lined trees within neighbourhoods could act as critical corridors for birds to move from one habitat to another [12]. In reality, specialized species cannot use all habitats present in a landscape [33,34,35]. Hence, there is a need for heterogeneity and presence of closely knit NPs could be an important landscape feature that vagile taxa rely on to extend their habitat requirement beyond just the local habitats. The number of habitat types is positively related to biodiversity across landscape [36]. Dramstad *et al*. (1996) argue that land cover diversity can contribute to biodiversity across different scales. Nielsen *et al*. (2013) reviewed 62 empirical research studies from 25 countries and pointed out that land cover heterogeneity may be the most important factor supporting urban biodiversity.

The results showed that there were equal proportion of native and exotic tree within NPs. Often exotics are perceived as a threat to biodiversity, but recent studies have revealed that non-native plant species could play a compensatory role in supporting native biodiversity especially in an urban landscape [37–41]. Also, exotic vegetation could be providing critical resources for native biodiversity especially during resource deficit period [41]. Hence, a mix of both native and exotic vegetation could help support biodiversity in an urban landscape.

Results also showed that birds in NPs are dependent on the presence of green spaces with a distance of 5 km, whereas butterflies and insects seem to be influenced by the immediate surroundings which comprise herb and shrub proportion. A combination of pocket green spaces along with avenue trees appears to contribute to structural variations in the landscape at the neighbourhood scale, which could help enhance the habitat requirements. The current habitat characteristics such as canopy cover, tree density for birds, herb and shrub proportion for butterflies and lawn proportion are influencing the biodiversity within NPs. These features within NPs, complemented with a green network at the immediate and larger surroundings, could influence local species richness at both the neighbourhood and city scales.

Growing cities seem to have an effect of decreasing, fragmenting and isolating natural patches by altering the size, shape and interconnectivity of natural landscapes [42]. In addition to physical expansion, a multitude of factors dealing with human activity can have a cascading effect such as a switch over from traditional to modern gardening practices, homogenization of landscapes and many more [43]. Bangalore city has diverse landscape configuration types and the most appropriate model that fits is the single large or several small theory (henceforth SLOSS) [44,45]. Interestingly, high density of NPs without the influence of a LGS (HNP–LGS) and low-density of NPs in the presence of a LGS (LNP +LGS), were found to support rich bird and butterfly diversity. Although, LGS have proven to support high biodiversity, this study indicates that several small green spaces can be a good representative of a single LGS. Even LGS within the city today are not essentially compact, witnessing development and are in fact patchy. Thus, several small NPs could supplement the large parks to function efficiently in conserving biodiversity within cities [46–49]. Besides, there is less scope to have large parks within city limits.

The fondness survey revealed that people are fond of butterflies and birds, and are less tolerant and/or intolerant towards various other taxa which they term ‘‘creepy crawlies”. Using a taxon which emerges at a high rank in the fondness survey as an ‘‘umbrella species” could motivate communities to alter their gardening practices, which in turn can also support many other taxa inadvertently. People’s tolerance towards biodiversity, could be influenced by lack of exposure to natural green spaces that support diverse taxa or could also be the effect of an individual’s gardening practices, resulting in poor biodiversity. For example, householders who still possess traditional gardens are likely to be more tolerant towards diverse taxa and adaptable to make changes to attract more biodiversity than individuals who are tolerant only towards selected species and prefer open landscape with lawn and a few flowering plants. Thus, any programme targeting enhancement of local species richness at neighbourhood scale, must acknowledge the diversity of attitudes arising either from historical or cultural perspectives [50] and understand how responsive landowners may be towards altering their activities to benefit biodiversity [51]. This study shows that management of birds and butterflies covers a range of habitats which is also suitable for other inconspicuous taxa. Also, the results show that birds are a good surrogate for insects. Evolving management practices that enhance cross-taxa congruence could not only bring back the inconspicuous taxa, but also build people’s tolerance towards them. Andersson *et al*. 2014 has demonstrated clearly that involvement of conservation stewards in urban biodiversity and ecosystem services, planning and governance can help improve the city green infrastructure immensely. Through citizen science projects around neighbourhood green spaces, there is provision for monitoring, which could serve as an important feedback on how household gardens contribute to biodiversity conservation within backyards. Efforts such as “Backyard Wildlife” are beginning to emerge even in India [52] and if the outputs are linked with actions on ground, it could help create a green neighbourhood with community involvement which will help bring back the traditional garden practices which supported biodiversity at the neighbourhood scale.

Contact with nature in the immediate surroundings is very critical [53], and many NPs offer such opportunities. However, studies to evaluate and measure connection to nature, have tended to focus on more ‘‘pristine” sites, nature as separate, as in ‘‘going to nature” rather than on what is immediately accessible [54]. Even NPs can support adequate biodiversity and decelerate the rapid rate of biodiversity loss; hence, these green spaces should no longer be viewed as depauperate ecosystems [55]. This study highlights that NPs in spite of their limited size, if connected through the development of green links could contribute significantly towards conserving biodiversity within the city. Projects involving community-based actions have shown that creating green infrastructure not only produces ecosystem services but also constitutes social-ecological processes that directly benefit human well-being [56].
